# Impact of respiratory motion on dose distribution in SIB‐SBRT for lung cancer

**DOI:** 10.1002/acm2.70136

**Published:** 2025-06-04

**Authors:** Lingling Liu, Zhenle Fei, Jie Li, Jiong Shu, Jingyuan Shao, Jianguang Zhang, Xiangli Cui, Hongzhi Wang

**Affiliations:** ^1^ University of Science and Technology of China Hefei China; ^2^ Hefei Cancer Hospital of CAS Institute of Health and Medical Technology, Hefei Institutes of Physical Science, Chinese Academy of Sciences Hefei China; ^3^ Department of Oncology The 901st Hospital of Joint Logistics Support Force of the Chinese People's Liberation Army Hefei China; ^4^ Department of Oncology Zibo Wanjie Hospital Zibo China

**Keywords:** 4D dose calculation, four‐dimensional CT, lung neoplasms, respiratory motion, SIB‐SBRT

## Abstract

**Purpose:**

Respiratory motion is a major source of dose uncertainty in lung cancer radiotherapy. The dose distribution of simultaneous integrated boost‐stereotactic body radiotherapy (SIB‐SBRT) is inhomogeneous and is significantly impacted by respiratory motion for lung cancer. The effect of respiratory motion on SIB‐SBRT was investigated with a four‐dimensional (4D) dose calculation method.

**Methods:**

Nineteen previously treated lung cancer patients were selected for this planning study. All patients underwent four‐dimensional CT (4D‐CT) scanning, and volumetric modulated arc therapy (VMAT) treatments were planned with internal target volume (ITV) and planning target volume (PTV). Dose distributions (3D‐plan) were calculated on the average reconstruction of the 4D‐CT. 4D dose distributions (4D‐plan) were calculated to evaluate respiratory motion effects. These calculations were performed on the CT images of related respiratory phase with a respiration‐correlated assignment of the 3D plan's monitor units to the respiratory phases of the 4D‐CT. Subsequently, the accumulative 4D dose based on deformable registrations of the CT series was generated and compared to the 3D dose distribution. Dosimetric deviations in targets and organs at risk (OARs) were analyzed with dosimetric parameters, and correlations between dose deviations (Δ*V*_100_ (ITV, PTV)) and patient characteristics (left–right, SI, anterior–posterior, S, L, Volume (ITV, PTV)) were explored.

**Results:**

With deformable registrations, the median values of relative differences between 3D‐plan and 4D‐plan 0 were found to be from −6.6% to 12.1% for all targets dosimetric parameters, and from −4.2% to 1.4% for OAR parameters. It was also shown that PTV coverage dropped more significantly than that of ITV with respiratory motion. Strong correlations were observed between the Δ*V*_100_ (ITV, PTV) and patient characteristic (SI, S, L).

**Conclusion:**

Respiratory motion effects during SIB‐SBRT treatment resulted in non‐negligible dose variability. Furthermore, with the correlation relationship and respiratory motion parameters, the dose coverage reduction of targets could be predicted.

## 1 INTRODUCTION

Lung cancer significantly contributes to cancer‐related morbidity and mortality worldwide.^1^ Due to its effectiveness, convenience, and non‐invasiveness, stereotactic body radiation therapy (SBRT) is currently the recommended treatment for medically inoperable lung cancer patients.^2^, ^3^ However, in clinical practice, although dynamic intensity‐modulated radiotherapy (IMRT) can improve radiation dose distribution and delivery to the surrounding crucial structures,^4^, ^5^, ^6^ patients with tumors near vital organs such as major blood vessels, the chest wall, trachea, or with poor pulmonary function often failed to tolerate definitive radiotherapy due to dose‐limiting toxicity. This may cause treatment interruptions or lead to inferior survival outcomes.^7^, ^8^

Thus, researchers have focused on developing an effective treatment regimen based on the simultaneous integrated boost (SIB) concept,^9^ which delivers a higher dose to the primary tumor volume and a relatively lower dose to the subclinical lesions. Therefore, the SIB‐SBRT strategy, which is capable of reducing radiation toxicity while maintaining local control, was chosen and applied in the treatment of certain lung cancer patients. Studies by Knight^10^ and Kenamond^11^ have shown that SIB‐SBRT is a safe, fast, and efficacious treatment for larger lung masses, offering promising local control rates and no adverse treatment‐related toxicity. Although Shen's study^12^ utilized the hypofractionated volumetric‐modulated arc radiotherapy (VMAT) combined with the SIB technique, with a lower fractional dose than the SIB‐SBRT technique, the results demonstrated favorable local control and survival with well‐tolerated toxicities.

However, the application of SIB‐SBRT to lung cancer is a more complicated scenario because respiratory‐induced tumor motion impacts target localization and also influences the accuracy of computed tomography (CT) imaging, as well as treatment delivery.^13^ Usually, respiratory motion affects the dose distribution in tumors in two ways: the blurring effect and the interplay effect.^14^ The effect of target motion on conformal radiotherapy can lead to an underdosage at the edges of the moving target volume, which is called the blurring effect. The internal target volume (ITV) concept, constructed using respiration‐correlated four‐dimensional CT (4D‐CT), ensures dose coverage of the tumor volume over a complete breathing cycle under free breathing conditions. When the dose is delivered homogeneously, it helps prevent underdosage at the tumor edges. However, because the dose gradient of SIB‐SBRT is larger within the ITV, the ITV concept is not fully capable of preventing the dose‐blurring effect. Furthermore, due to dynamic treatment techniques, tumor movement may interact with the dynamic radiation field, known as the interplay effect.^15^ This effect can also result in differences between the planned dose and the delivered dose. These dose variations depend on specific treatment techniques and patient‐specific parameters,^14^ such as respiratory rate, amplitude of organ motion, and asymmetry of the respiratory cycle.^16^ Although Bortfeld^14^ demonstrated that the interplay effect is negligible after a long series of fractions, it remains a concern in SIB‐SBRT due to the hypofractionated nature of the treatment. In contrast to conventionally fractionated treatments, multiple studies^17^, ^18^, ^19^ have shown that the dosimetric impact of the interplay effect is more pronounced in hypofractionated treatments.

To date, the potential impact of the interplay effect and the inhomogeneously planned dose of SIB‐SBRT treatments within the ITV has not been studied by other researchers in the field of treatment planning. Therefore, based on the phase‐sorted respiration‐correlated 4D‐CT image sets, a 4D dose calculation method was used to investigate the dose deviations for ITV and planning target volume (PTV). Additionally, the correlation factors leading to the dose variation are also investigated in this paper.

## 2 MATERIALS AND METHODS

### 2.1 Patients and image acquisition

Nineteen patients with lower lobe (*n* = 5), upper lobe (*n* = 8), and middle lobe (*n* = 6) lung cancer were treated with SIB‐SBRT. For each patient, a 4D‐CT image set was acquired using a Philips Big Bore 16‐slice CT simulator (Philips Healthcare, Amsterdam, Netherlands) with a 4D retrospective pulmonary gating protocol (120 kVp, 1 mm slice thickness, collimation 16 × 1.5, standard resolution, pitch 0.079, rotation time 0.5 s, field of view (FOV) 500 mm, and a standard filter (B)) with a phase sorting algorithm. All patients were positioned with vacuum cushions and scanned in a head‐first supine position. A bellow was placed across the patient's chest to measure changes in lung volume. Patients did not receive specific breathing instructions prior to the CT scanning.

### 2.2 Treatment planning

The 4D‐CT images were imported into the Monaco treatment planning system (TPS) (Monaco 5.11, Elekta, Sweden). The averaged intensity projection (AIP) images and maximum intensity projection (MIP) were generated from the 4D‐CT data. Subsequently, the ITV was delineated on the AIP image using the MIP image as a reference, with additional corrections based on visual inspection of all 10 reconstructed breathing phases. An isotropic margin of 5 mm was added around ITV to create PTV.

The prescription dose was 50 Gy in 10 fractions to the PTV and 60 Gy in 10 fractions to the ITV, delivered using a VMAT technique in the SIB‐SBRT plan. The SIB‐SBRT plan (termed “3D‐plan”) was designed on an Infinity linear accelerator (LINAC) (Elekta, Sweden) equipped with a 160‐leaf multi‐leaf collimator (MLC). The dose distributions were calculated using 6 MV photons with the Monte Carlo algorithm implemented in the Monaco TPS. The dose calculation was performed in a single arc, with a dose grid resolution of 1 mm × 1 mm × 1 mm. The minimum segment width was 5 mm, and the statistical uncertainty was set to 1%.

### 2.3 Target motion

To investigate the relationship between target motion and target dose discrepancies, the target motion amplitude was quantified for all patients. Based on the 4D‐CT series, the amplitude of the target motion was defined as the maximum coordinates minus the minimum coordinates of the gross target volume (GTV) centroid in three directions (left–right (LR), superior–inferior (SI), and anterior–posterior [AP]). The 3D scalar amplitude (termed “L”) was calculated as L = $\;\sqrt{LR^{2}+SI^{2}+AP^{2}}$. For each patient, the LR, SI, AP, and L values are listed in Table 1.

**TABLE 1:** Patients characteristics.

Patient#	ITV volume\cm^3^	LR\cm	SI\cm	AP\cm	L\cm	S\cm
**1**	6.9	0.1	2.7	0.3	2.7	1.9
**2**	1.5	0.1	0.8	0.4	0.9	3.9
**3**	2.7	0.2	2.1	0.1	2.1	1.7
**4**	3.5	0.3	0.3	0.3	0.5	13.8
**5**	11.6	0.2	1.2	0.3	1.3	1.7
**6**	17.8	0.3	0.5	0.2	0.6	4.1
**7**	5.2	0.1	0.2	0.3	0.3	17.6
**8**	10.7	0.3	0.5	0.2	0.6	10.9
**9**	3.7	0.6	0.6	0.4	0.9	5.6
**10**	36.2	0.2	0.6	0.2	0.7	4.7
**11**	6.9	0.1	0.6	0.4	0.7	7.2
**12**	58.0	0.4	1.5	0.6	1.7	3.3
**13**	11.6	0.2	1.2	0.3	1.3	6.2
**14**	11.0	0.2	0.6	0.3	0.7	4.1
**15**	8.3	0.1	0.0	0.2	0.2	5.4
**16**	15.8	0.3	0.3	0.5	0.6	7.5
**17**	15.8	0.3	0.6	0.2	0.7	3.0
**18**	16.2	0.2	0.2	0.2	0.3	14.1
**19**	1.1	0.3	0.5	0.5	0.7	5.8

The diaphragm is a respiratory muscle that accounts for approximately 80% of all respiratory work in normal tidal breathing.^20^ For all nineteen patients, the minimum distance from the ITV centroid to the diaphragm (termed “S”) were also listed in Table 1.

### 2.4 Description of the 4D dose calculation method

**FIGURE 1:**
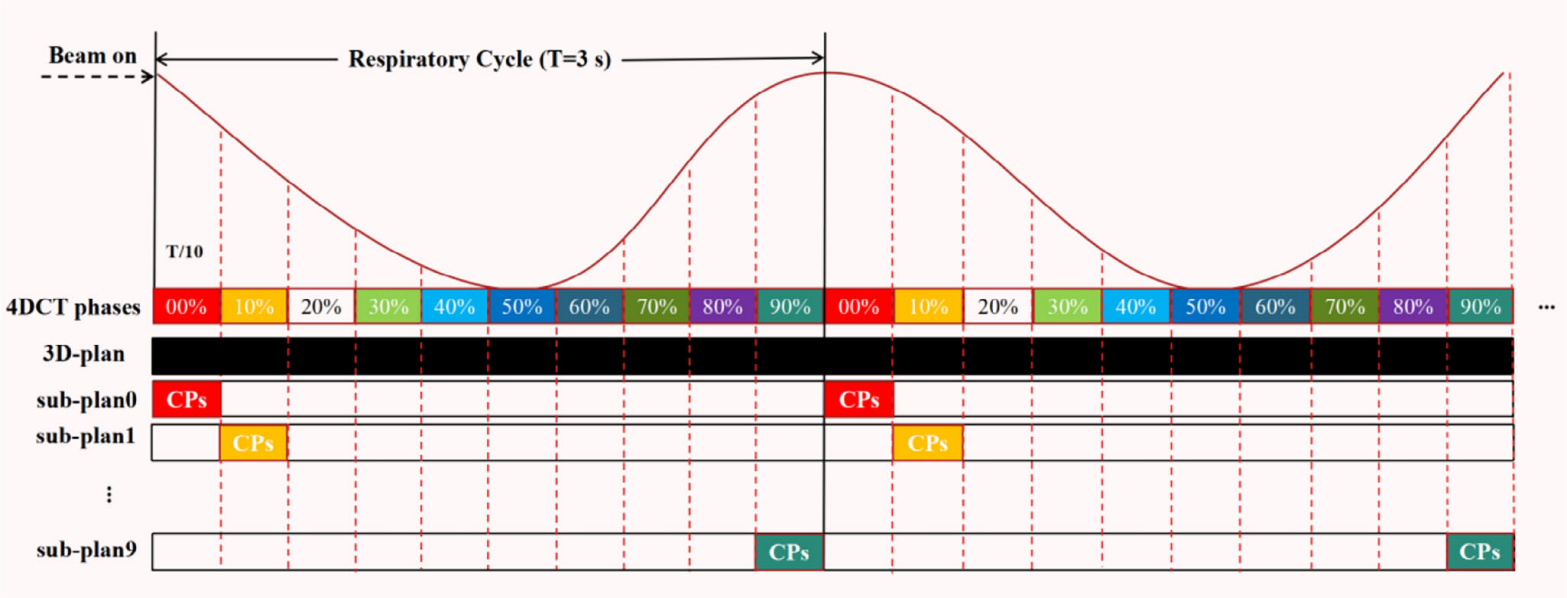
The respiratory cycle was divided into 10 equally long phases. The CPs of the 3D‐plan were assigned to the dedicated respiration phases of 4D‐CT series, respectively. Then, 10 phase‐specific sub‐plans were calculated. 4D‐CT, four‐dimensional CT; CP, control point.

For each patient, after the delivery of one fraction, the machine delivery log file, which recorded the time stamps for the control points (CPs) and corresponding monitor units (MUs), was collected. Based on the findings of Ehrbar et al. ,^21^ a regular respiratory cycle of 3.0 s was assumed for all patients. Using the delivery log file and respiratory cycles (termed “T”), the time section corresponding to each respiratory phase was determined. Subsequently, the segment CPs and MUs were assigned to the 10 respiratory phases of the 4D‐CT. Finally, all CPs falling within the same respiration phase were used to calculate the dose for that phase, creating what we termed a “sub‐plan.” Figure 1 shows the CPs and MUs of the 3D‐plan in their chronological assignment to the dedicated respiration phases. Therefore, 10 phase‐specific sub‐plans were generated.

**FIGURE 2:**
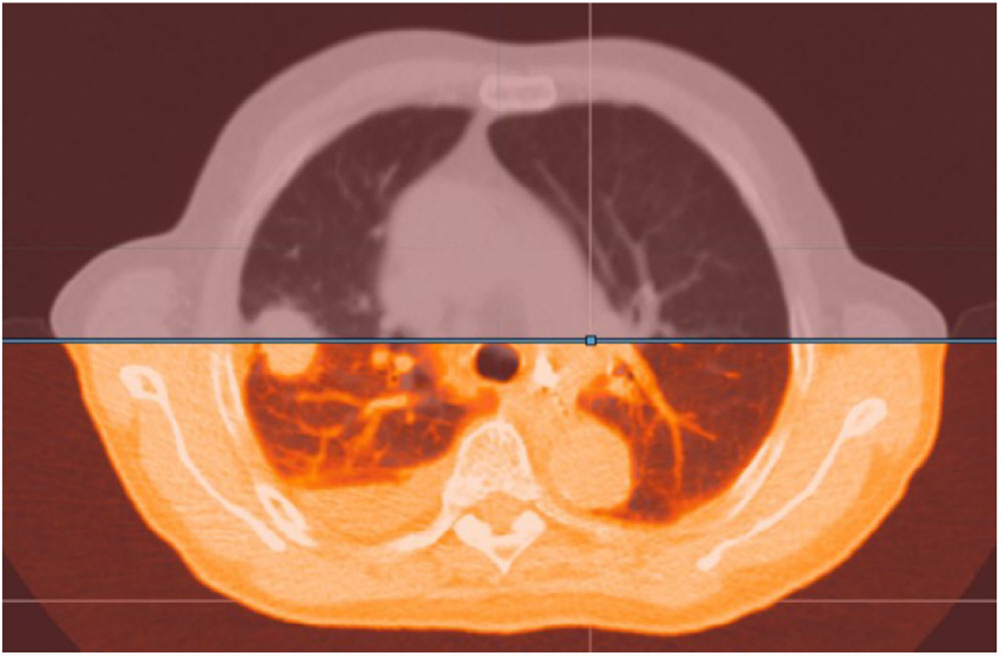
Result after deformable registration for AIP CT series and one single‐phase CT series. AIP, averaged intensity projection; CT, computed tomography.

To evaluate the dose variations in the moving ITV and PTV, 4D dose accumulations were performed with AccuContour software (Xiamen Manteia Technology Ltd., China). First, the AIP CT series and a single‐phase CT series were selected. The built‐in deformable image registration tools based on the Demons algorithm were employed to perform deformable registrations from the single‐phase CT series onto the AIP CT series, and Figure 2 shows the result after deformable registration for AIP CT series and one single‐phase CT series. Meanwhile, the dose of the single‐phase CT series was deformed termed the transferred dose. The same procedure was performed for the other nine single‐phase CT series, and nine transferred dose could be obtained. Finally, the deformable dose accumulations on the AIP CT series^22^, ^23^, ^24^ were obtained by summing the 10 transferred dose. This accumulation was termed “4D‐plan 0,” including variation in HU, interplay effect and deformable registration effect.

### 2.5 Assessment of the impact of respiratory motion on dose distribution

To quantify the effect of respiratory motion on dose coverage of target volumes, dose‐volume histogram (DVH) metrics for the target volumes (ITV and PTV) in the 3D‐plan were compared with those in the 4D‐plan 0. The following dosimetric parameters were recorded: *D*_95_, defined as the least doses received by 95% of the target volume; *V*_100_, defined as the volume of the target receiving 100% of the prescribed dose; maximum dose (*D*_max_); mean dose (*D*_mean_); minimum dose (*D*_min_); heterogeneity index (HI)^25^; conformity index (CI)^26^, ^27^; and gradient index (GI).^28^

Meanwhile, dosimetric parameters were recorded to evaluate the dose variations for organs at risk (OARs) between the 3D‐plan and the 4D‐plan 0. For the lungs, parameters included the volumes receiving 5 Gy (*V*_5_), 10 Gy (*V*_10_), 20 Gy (*V*_20_), and 30 Gy (*V*_30_), as well as the *D*_mean_. For the heart, parameters were the *D*_max_ and the *D*_mean_. For the chest wall, the *D*_max_ was recorded. For the spinal cord, the *D*_max_ was assessed.

The relative difference in dose distribution between 3D‐plan and 4D‐plan 0 was mainly caused by the variation in HU (between AVG CT and 10 phase CTs), interplay effect and deformable registration. To evaluate the contributions of these effects, we calculated each effect on the dose distribution with the 4D dose calculation method. First, the temporal effects were neglected, and the MU of every segment had to be distributed uniformly to the 10 breathing phases, then the 10 phase‐specific sub‐plans were generated for the 10 respiratory phases, each with 10% of the MU from the 3D‐plan. The resulting 4D dose accumulations (termed “4D‐plan 1,” including variation in HU) were obtained without performing deformable registration. Second, in order to assess the contribution of interplay effect, a 4D dose accumulation (termed “4D‐plan 2,” including variation in HU, the interplay effect) was generated without performing deformable registration, based on 10 phase‐specific sub‐plans created using the 4D dose calculation method. Notably, the sub‐plans used to produce 4D‐plan 1 and 4D‐plan 2 were generated in different way. Meanwhile, the dose deviation between 4D‐plan 1 and 4D‐plan 2 caused by the interplay effect was calculated. Similarly, the dose deviation between 4D‐plan 0 and 4D‐plan 2 could be calculated, representing the contribution of deformable registration.

For nineteen patients, dosimetric parameter values from both 3D‐plan and 4D‐plan 0 were underwent statistical analysis using the Wilcoxon signed‐rank test. Statistical analysis was performed using SPSS v25.0 (International Business Machines Corp., Armonk, New York, USA). Both the dosimetric parameters and their relative differences of nineteen patients were shown as medians with interquartile ranges (IQRs). A *p*‐value of less than 0.05 (*p* < 0.05) was considered statistically significant.

To explore the reasons leading to the target dose deviation, correlation studies were performed using Spearman's correlation coefficients to analyze the relationship between the dose deviation of targets (Δ*V*_100_) and patient characteristics. A *p*‐value of less than 0.05 (*p* < 0.05) was considered statistically significant.

## 3 RESULTS

### 3.1 Effect of respiratory motion on targets and OARs

ITV and PTV were the tumor targets. The dosimetric parameters of these targets were evaluated for both the 3D‐plan and the 4D‐plan 0. Additionally, for each dosimetric parameter, the relative difference between the 3D‐plan and the 4D‐plan 0 was analyzed. Table 2 presents the comparative results for ITV and PTV. Except for the HI of ITV, all other dosimetric parameters showed statistical significance, indicating that respiratory motion had a significant effect on the dose distribution within the target volumes. Furthermore, compared to the 3D‐plan results, the results of 4D‐plan 0 were equal to or lower for all the dosimetric parameters of the targets.

**TABLE 2:** Comparison of dosimetric parameters of targets (ITV and PTV) between 3D‐plan and 4D‐plan 0.

Targets	Dosimetric parameters	3D‐plan	4D‐plan 0	Relative difference %	*p*‐value
**ITV**	*V*_100_ (%)	98.8(1.2)	97.0(5.1)	−1.6(4.7)	0.002
	D_95_ (Gy)	61.1(0.5)	60.5(1.1)	−1.0 (2.4)	0.001
	*D*_min_ (Gy)	58.0(2.4)	56.0(3.3)	−1.7 (4.6)	0.024
	*D*_max_ (Gy)	68.5(2.3)	66.3(1.8)	−3.2 (1.0)	< 0.001
	*D*_mean_(Gy)	63.8(1.2)	63.1(1.2)	−1.0(1.0)	< 0.001
	HI	1.1(0.0)	1.1(0.0)	0.0(2.1)	0.549
	CI	0.6(0.2)	0.7(0.2)	11.8(13.1)	0.002
	GI	11.5(10.2)	10.9(10.4)	−2.8(3.3)	< 0.001
**PTV**	*V*_100_ (%)	98.3(1.8)	93.8(4.9)	−3.5 (4.2)	< 0.001
	D_95_ (Gy)	52.2(0.9)	49.4(2.4)	−4.2 (4.0)	< 0.001
	*D*_min_ (Gy)	43.2(6.5)	38.4(5.4)	−6.4(13.7)	0.001
	*D*_max_ (Gy)	68.5(2.3)	66.3(1.8)	−3.3(1.2)	< 0.001
	*D*_mean_(Gy)	60.0(1.2)	58.4(0.6)	−2.2 (2.0)	< 0.001
	HI	1.3(0.1)	1.3(0.1)	3.2(4.1)	< 0.001
	CI	0.8(0.2)	0.8(0.2)	3.5(5.3)	0.022
	GI	5.8(2.2)	5.5(2.5)	−4.1(2.9)	< 0.001

*Note*: The data are presented as median (IQR) values.

Meanwhile, the impact of respiratory motion on the dose distributions of OARs was analyzed and is summarized in Table 3. Except for the *D*_mean_ of the heart, all other dosimetric parameters showed statistically significant differences. Additionally, except for the *V*_5_ of the bilateral lungs, all other dosimetric parameters of 4D‐plan 0 were not higher than those parameters of the 3D‐plan.

**TABLE 3:** Comparisons of dosimetric parameters of OARs between 3D‐plan and 4D‐plan 0.

OARs	Dosimetric parameters	3D‐plan	4D‐plan 0	Relative differences %	*p*‐value
**Bialteral Lungs**	*V*_5_ (%)	16.5(10.3)	16.9(10.5)	−1.2(1.8)	0.003
	*V*_20_ (%)	5.8(5.1)	5.6(4.8)	−1.8(2.8)	0.004
	V_30_ (%)	3.0(2.3)	3.0(2.3)	−2.1(4.0)	< 0.001
	*D*_mean_ (Gy)	3.9(2.4)	3.9(2.3)	−0.7(0.9)	0.007
**CW**	*D*_max_ (Gy)	53.8(29.2)	51.7(27.7)	−3.2(3.1)	0.002
**Heart**	*D*_max_ (Gy)	18.6(27.2)	18.0(24.3)	−3.7(7.7)	0.001
	*D*_mean_ (Gy)	2.3(4.0)	2.3(4.0)	0.0(1.2)	0.856
**Spinal cord**	*D*_max_ (Gy)	9.1(8.9)	8.8(8.5)	−4.2(2.4)	< 0.001

*Note*: The data are presented as median (IQR) values.

**FIGURE 3:**
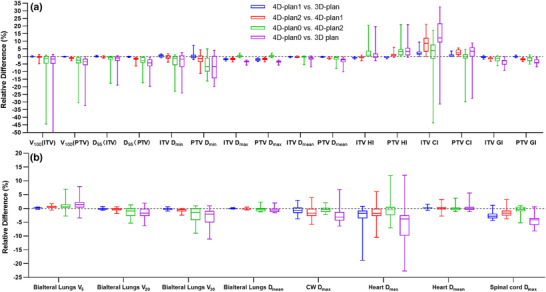
The median value of relative difference for dosimetric parameters. Blue, red, and green marks represent the median value of relative difference (dosimetric parameters) caused by variation in HU, interplay effect, deformable registration effect, respectively. Purple represents the total effect (including variation in HU, interplay effect, and deformable registration effect).

The contributions of variation in HU, interplay effect and deformable registration were evaluated and shown in Figure 3, respectively. For the contribution of variation in HU, the median values of the relative difference were less than 1%. Meanwhile, the relative differences in dosimetric parameters between 4D‐plan 1 and 4D‐plan 2 caused by the interplay effect were less than 1.7%, except for CI (CI_ITV _= 8.6%, CI_PTV _= 2.4%). However, for the contribution of deformable registration, most of median values were less than 4%, which accounts for most of the total contribution.

### 3.2 Correlation between target dose deviation and patient characteristics

In this study, the correlations between Δ*V*_100_ (ITV, PTV) and patient characteristics (LR, SI, AP, S, L, Volume (ITV, PTV)) were analyzed and are shown in Table 4. The Δ*V*_100_ (ITV, PTV) showed strong correlations with patient characteristics (SI, L, S). Moreover, the correlation coefficients between the Δ*V*_100_ and patient characteristics (SI, L, S) were statistically significant. In contrast, no significant correlations were observed between Δ*V*_100_ (ITV, PTV) and other patient characteristics (LR, AP, Volume (ITV, PTV)).

**TABLE 4:** The correlation between target dose reduction and patient characteristics.

	Spearman's correlation coefficients (*r*, *p*‐value)	
	Δ*V*_100_ (ITV)	Δ*V*_100_ (PTV)
**Volume (ITV)**	(−0.002, 0.994)	(0.135, 0.581)
**Volume (PTV)**	(−0.012, 0.960)	(0.111, 0.652)
**LR**	(0.081, 0.742)	(0.288, 0.232)
**SI**	(0.861, < 0.0001)	(0.691, 0.001)
**AP**	(0.268, 0.268)	(0.414, 0.078)
**L**	(0.903, < 0.0001)	(0.777, < 0.0001)
**S**	(−0.812, < 0.0001)	(−0.625, 0.004)

**FIGURE 4:**
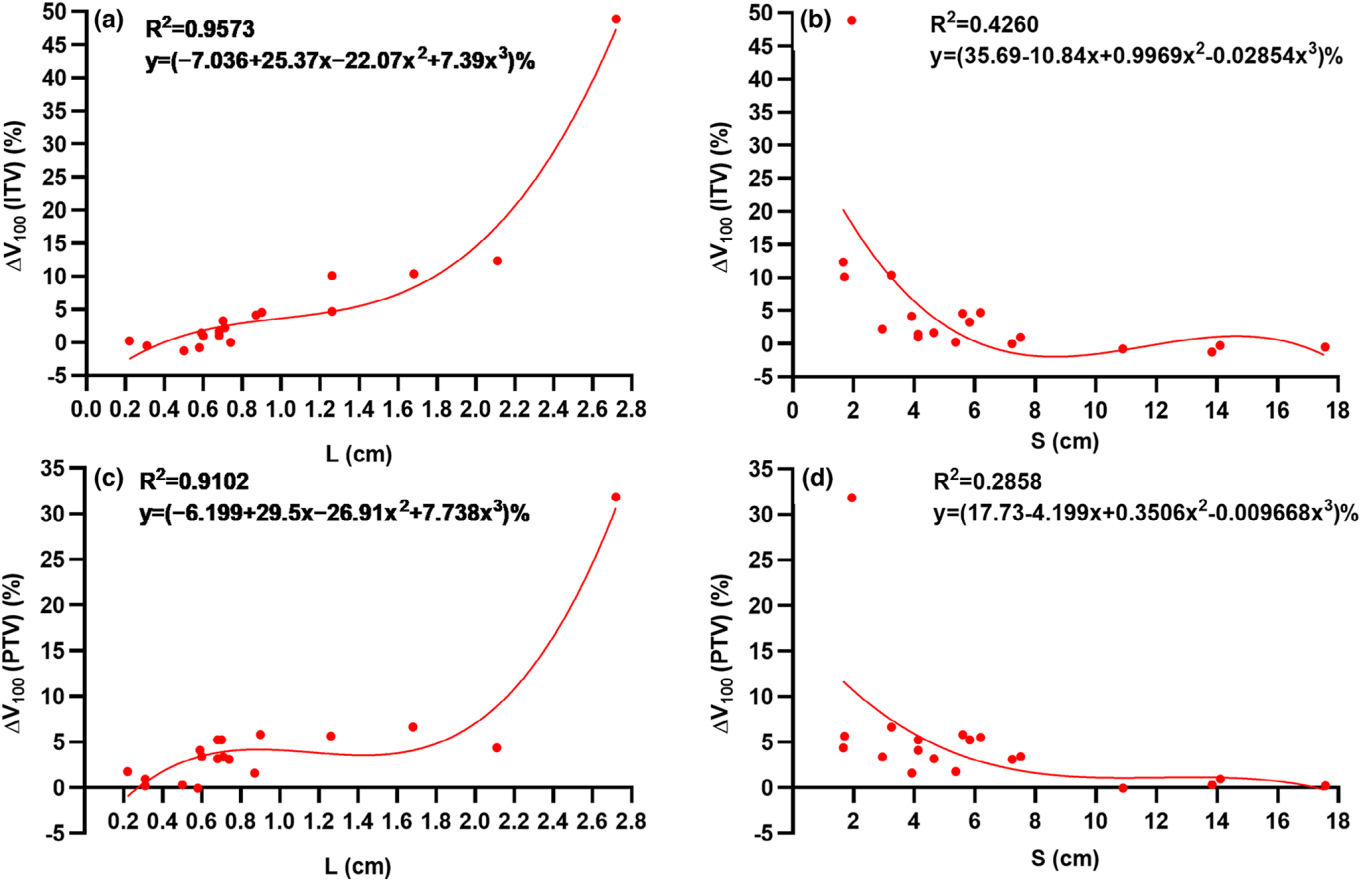
The correlations between Δ*V*_100_ (ITV, PTV) and patient characteristics (L, S) were fitted with a polynomial function. (A) and (C) show the fits for Δ*V*_100_ (ITV, PTV) and L. (B) and (D) show the fits for Δ*V*_100_ (ITV, PTV) and S. ITV, internal target volume; L, 3D scalar amplitude; PTV, planning target volume; S, minimum distance from the ITV centroid to the diaphragm.

To extract the quantitative correlation between Δ*V*_100_ (ITV, PTV) and patient characteristics (L, S), a polynomial function (*y *= *B*_0_ + *B*_1_ *x* + *B*_2_ *x* ^2^ + *B*_3_ *x* ^3^) was used to fit the data, the fitting results are shown in Figure 4. With the fitting functions and the values of L or S, the dose deviation (Δ*V*_100_) caused by respiratory motion can be predicted in clinical practice. Compared with ITV, the polynomial fitting with the S parameter showed a weaker correlation for PTV.

## 4 DISCUSSION

In clinical practice, for lung cancer patients whose tumors are located near critical structures and who fail to tolerate definitive radiotherapy, the SIB‐SBRT technique is chosen. However, during the implementation of SIB‐SBRT in lung cancer, the effects of respiratory motion and the dynamic components of the linear accelerator on dose distribution must be carefully considered. Therefore, in this study, we performed a 4D‐CT planning study to investigate the extent of motion effects on the dose distribution caused by dynamic dose delivery of VMAT and respiratory motion for an inhomogeneous SIB‐SBRT treatment plan.

**FIGURE 5:**
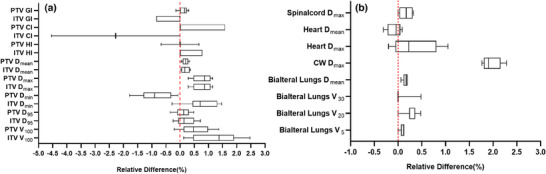
With nine different initial phases with beam‐on timing, the relative difference of dosimetric parameters was calculated. (a) and (b) are the relative differences of dosimetric parameters for targets and OARs, respectively. OAR, organs at risk.

In this paper, based on phase‐sorted 4D‐CT image series, a 4D dose calculation method inspired by Ehrbar et al.^21^ was developed to calculate the effective dose distribution for the patient. Here, the 00% phase was supposed as the starting phase. To investigate the impact of the chosen starting phase, the patient with the largest 3D scalar amplitude was selected, and the corresponding 4D‐plan was recalculated repeatedly using nine different initial phases determined by beam‐on timing. The relative differences in dosimetric parameters were calculated for targets and OARs, as shown in Figure 5. For all the dosimetric parameters, their relative differences were averaged, resulting in an average of 0.5%. Similar results were obtained in the studies of Roberts^29^ and Ehrbar.^21^ Therefore, it was considered reasonable to assume the 00% phase as the starting phase.

**FIGURE 6:**
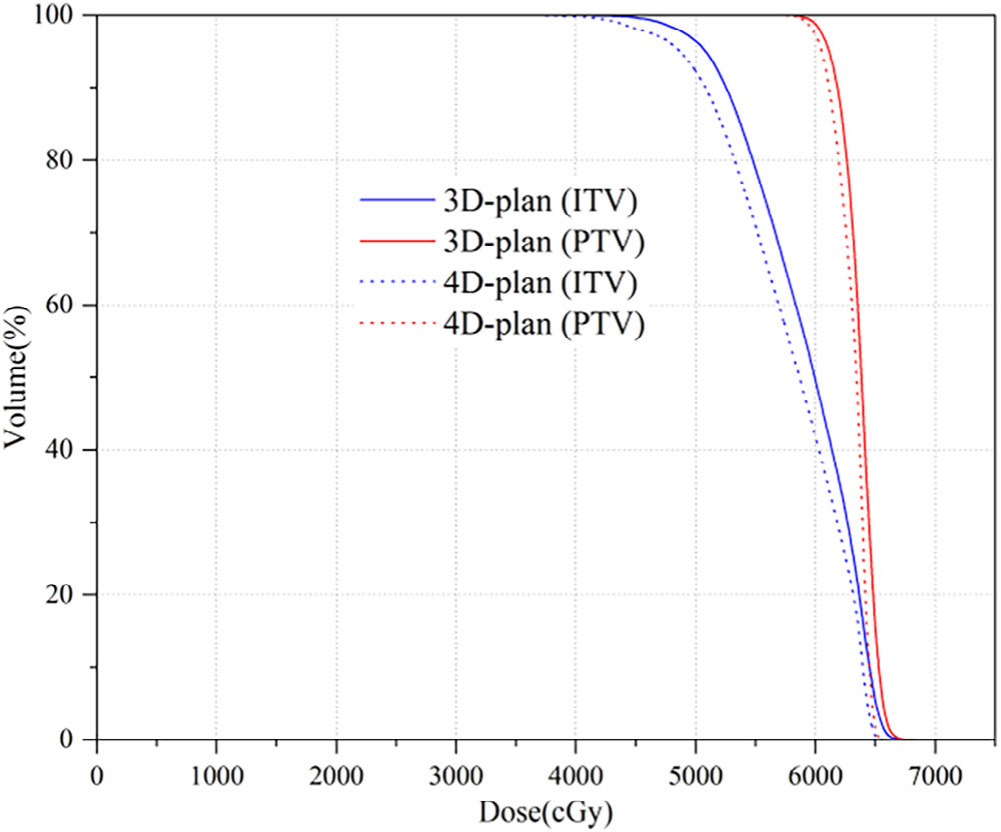
DVH comparisons between 3D‐plan and 4D‐plan 0 dose distributions for a patient with respiratory motion (L = 0.6 cm). DVH, dose‐volume histogram.

With the 4D dose calculation method, the effect of respiratory motion on ITV, PTV, and OARs was evaluated. We found that almost all the relative differences in dosimetric parameters were statistically significant. However, compared to the ITV, the dosimetric parameters were more severely affected for PTV. Figure 6 shows DVH comparisons between the 3D‐plan and 4D‐plan 0 for a patient with a respiratory motion (L = 0.6 cm), and the comparison results demonstrate that the PTV was affected seriously. Li^30^ investigated the interplay effect in VMAT‐based lung SBRT, and DVH comparisons were made between 3D and 4D dose distributions for a patient with a respiratory motion of 1.6 cm. These DVH comparisons showed that the coverage of the PTV volume dropped significantly compared to the GTV or GTV + 5 mm, which was similar to our results.

In this study, the contributions of variation in HU, interplay effect and deformable registration were evaluated with the 4D dose calculation method. Based on the analyzed data, the contribution of dose deviation caused by variation in HU was the smallest. However, the spinal cord (*D*_max_) had the largest median value (−2.8%), suggesting that the *D*_max_ of spinal cord should be carefully monitored and kept within a safe limit. Both the interplay effect and deformable registration had a significant impact on the dosimetric parameters, therefore, both effects warrant careful consideration.

In order to explore the main influencing factors for target dose deviation, we did a correlation study. Three factors (L, SI, S) were found to have a strong correlation with Δ*V*_100_. However, SI accounts for about 65% of L. Therefore, we only conducted a quantitative correlation analysis between Δ*V*_100_ (ITV, PTV) and patient characteristics (L, S). With the same polynomial function, the fitting results were better for the L parameter compared to those for the S parameter. We performed a detailed analysis of the fitting data and results. First, patients (No.1, 3, and 5) had larger 3D scalar amplitudes, smaller tumor volumes, and tumors located in lower lobes, resulting in Δ*V*_100_ (ITV) values that were significantly higher (48.9%, 12.3%, and 10.1%, respectively) than the mean value (5.43%) averaged across nineteen patients. Second, for the L parameter, patients (No. 1, 3, and 5) also exhibited larger differences in Δ*V*_100_ (ITV) between measured data and values calculated using the fitting function—specifically, 1.5%, 5.3%, and 5.5%, respectively. Meanwhile, for the S parameter, these differences were even more pronounced (30.7%, 8.0%, and 9.9%, respectively), which were much larger than those obtained for the L parameter. Therefore, this difference may indicate that the parameters L and S have different associations with Δ*V*_100_ (ITV). Similar results were observed for PTV. Subsequently, based on the 5% deviation of clinical dose and the correlation fitting function, we calculated the maximum permissible target motion amplitude (L = 1.3 cm) and the minimum distance from the ITV centroid to the diaphragm (S = 4.5 cm).

## 5 CONCLUSION

In this work, a study was performed to investigate the differences in dose distribution between 3D‐plan and 4D‐plan for SIB‐SBRT treatment. The respiratory motion effects during SIB‐SBRT treatment resulted in non‐negligible dose variability. Furthermore, targets dose reduction due to respiratory motion could be predicted based on the established correlation.

## AUTHOR CONTRIBUTIONS

**Lingling Liu**: Writing original draft, performed the analysis. **Zhenle Fei**: Interpreted the data. **Jie Li**: Collected the logfile data. **Jiong Shu**: Collected the 4DCT data. **Jingyuan Shao**: Collected the 4DCT data. **Jianguang Zhang**: Interpreted the data. **Xiangli Cui**: Writing review and editing. **Hongzhi Wang**: Writing review and editing.

## ETHICS STATEMENT

This study was approved by the Ethics Committee of Hefei Cancer Hospital, Chinese Academy of Sciences (PJ‐KYSQ2024‐008). All methods were carried out in accordance with relevant guidelines and regulations.

## CONFLICT OF INTEREST STATEMENT

The authors declare no conflicts of interest.

## Data Availability

Requests for data access may be directed to the corresponding author.
